# Effect of Rising Temperature on Lyme Disease: *Ixodes scapularis* Population Dynamics and *Borrelia burgdorferi* Transmission and Prevalence

**DOI:** 10.1155/2019/9817930

**Published:** 2019-09-16

**Authors:** Dorothy Wallace, Vardayani Ratti, Anita Kodali, Jonathan M. Winter, Matthew P. Ayres, Jonathan W. Chipman, Carissa F. Aoki, Erich C. Osterberg, Clara Silvanic, Trevor F. Partridge, Mariana J. Webb

**Affiliations:** Dartmouth College, Hanover, NH 03755, USA

## Abstract

Warmer temperatures are expected to increase the incidence of Lyme disease through enhanced tick maturation rates and a longer season of transmission. In addition, there could be an increased risk of disease export because of infected mobile hosts, usually birds. A temperature-driven seasonal model of *Borrelia burgdorferi* (Lyme disease) transmission among four host types is constructed as a system of nonlinear ordinary differential equations. The model is developed and parametrized based on a collection of lab and field studies. The model is shown to produce biologically reasonable results for both the tick vector (*Ixodes scapularis*) and the hosts when compared to a different set of studies. The model is used to predict the response of Lyme disease risk to a mean annual temperature increase, based on current temperature cycles in Hanover, NH. Many of the risk measures suggested by the literature are shown to change with increased mean annual temperature. The most straightforward measure of disease risk is the abundance of infected questing ticks, averaged over a year. Compared to this measure, which is difficult and resource-intensive to track in the field, all other risk measures considered underestimate the rise of risk with rise in mean annual temperature. The measure coming closest was “degree days above zero.” Disease prevalence in ticks and hosts showed less increase with rising temperature. Single field measurements at the height of transmission season did not show much change at all with rising temperature.

## 1. Introduction

Lyme disease is the most common vector-borne disease in the US and accounts for 82% of reported tick-borne cases [1]. It is characterized by an initial infection by the spirochete *B. burgdorferi* which, if left untreated, can lead to severe consequences later [1, 2]. Lyme disease has two primary vectors: the black-legged tick (*I. scapularis*), found throughout the Eastern and North Central United States, with infectious ticks most abundant in the Northeast [3] and the western black-legged tick (*Ixodes pacificus*), found along the Pacific coast. Here, we focus on *I. scapularis.*

Because temperature controls many aspects of the tick life cycle, many efforts have been made to understand the dependence of *I. scapularis* life cycle on temperature [4–9] and to link climate to the potential range of this vector [10–16]. The mechanisms underlying the link between temperature and tick abundance can be approached via models such as the one developed here, as well as others [5, 7–9]. It is widely claimed that an increase in overall temperature will affect tick-borne diseases in general [17–21] and Lyme disease in particular has been the subject of a few models based on various measures of risk [10, 11, 16, 22–24].

Only a few dynamic models of Lyme disease transmission exist. Ogden et al. use their temperature-dependent life cycle model to model transmission between ticks and mice [25]. A model by Gaff and Gross for a different tick-borne disease combines all hosts into one population and does not track the full vector lifecycle. It does incorporate a version of seasonality and extends the model to multiple patches [26].

This study introduces a model of both tick life cycle and Lyme disease transmission based on the work of Ogden et al. [5, 6]. In Ogden et al.'s [5] study, a model of the tick life cycle is presented in which temperature is incorporated via a time delay and host densities are not modeled directly but represented by varying the probability that a questing tick finds a host. No disease is modeled. In [25], this model was extended to include disease transmission and a single host, *Peromyscus leucopus*, whose life cycle was modeled in some detail. In this study, we simplify the *P. leucopus* life cycle but extend the model to multiple host classes with different transmission dynamics, tick removal capacities, and population densities.

The most straightforward measure of disease risk to humans is the number of infected ticks questing for a blood meal in a given area. An increase in this abundance translates directly to an increased probability of contact with humans [3]. However, tick abundance is difficult to measure outright in the field and an estimate must be provided through a model such as the one developed here.

The most commonly used measure of risk is the number of degree days above 0 Celsius, which is an indicator of temperature suitability for *I. scapularis*, both at present [10] and under conditions of rising temperature [22, 27–31]. Other factors besides temperature have been used to indicate habitat suitability as well [24]. Neither habitat nor climatological suitability alone provides a robust estimate of the density of ticks present, as this depends also on host distribution and whether ticks are recently introduced or long established, as well as the effect of temperature on maturation rates of larvae and nymphs.

A common field measurement is the prevalence of infection in questing ticks [21, 32, 33], which varies depending on location and time of year. Although useful, it is an incomplete measure of risk without accompanying tick population data.

A useful proxy for risk to humans could be disease prevalence in host animals. A rise in this prevalence would indicate either more ticks or higher infection rates in ticks, and either way could be an indicator of increased disease risk to humans. *B. burgdorferi* rates in mice have been measured in some studies, which also observe a seasonal trend [34, 35].

Some species of birds are competent and highly mobile hosts for Lyme disease and have been considered a source of introduction of the disease to new areas [10]. Disease prevalence in this host represents a different type of risk: the risk of exporting the disease elsewhere.

The model developed here represents a synthetic ecosystem with disease dynamics present that is complete enough to consider all of the risk measures mentioned, not only for a single example but also under conditions of rising temperature. Taken together, these risk measures give a picture of the likely effect of a rise in mean annual temperature on risk of Lyme disease in regions similar to the Northeastern United States.

## 2. Materials and Methods

This study is based on the work of Ogden et al. which includes laboratory and field experiments as well as models [5, 6]. Compartment models of ordinary differential equations describe both the *I. scapularis* population, at various life stages, and the host populations to model the disease dynamics within a one-square kilometer area. The model of Ogden et al. was revised to be implemented using the Matlab ODE solver [36] and extended to include more host categories than the original model. Laboratory experiments have shown a clear temperature dependence of maturation periods for *I. scapularis* [5]. These temperature-sensitive rates are incorporated into this model to produce recognizable seasonal patterns of questing activity, based on the seasonal cycle of 2-meter air temperature for Hanover, NH. Humidity also plays a role in measured tick abundance [37, 38], but given the relatively high levels of warm season precipitation in Hanover, NH, we assume humidity is not limiting.

Ticks feed on a wide range of mammal hosts, from mice and other rodents to larger mammals such as raccoons and deer, as well as nonmammal hosts such as birds and reptiles. Some of these tick hosts such as *P. leucopus* [4, 25] are competent hosts for *B. burgdorferi* and serve as a reservoir from which uninfected ticks can pick up the disease, while others are incompetent hosts that play a role in propagation of the *I. scapularis* population [39, 40] through providing blood meals but are incapable of transmitting the disease. Although mice have been strongly implicated in the prevalence of both the black-legged tick and the transmission of the Lyme disease spirochete, it is acknowledged that other hosts play a role in Lyme disease transmission [2, 39, 41, 42]. In particular, mobile hosts, such as birds and deer, can carry both the vector and the disease to new locations, where it can become endemic [10]. Consequently, in this study, we have six classes of host: incompetent mobile, incompetent stationary, competent mobile (infected and uninfected), and competent immobile (infected and uninfected). The full six-host model enables us to compare the model output with a wide range of field observations.

Although the various species of host in a given category may vary in competence, we assume the hosts in the incompetent categories could not transmit disease at all. The probability of transmission upon contact with an infected tick was calculated as a weighted average over all the species in a competent host category, based on rates reported in Levi et al. [39].

Birth, death, and maturation rates are based on measurements in both the laboratory and field. Densities of host populations and ticks per host are based on published studies, with references provided in Table 1. In particular, our single patch model includes six host populations categorized by competence, infectious versus uninfected, and mobile versus immobile. However, no published data set includes complete information on all stages of the tick lifecycle throughout a season, tick densities on various hosts, and host densities in the landscape.

Many sources were used to estimate parameters in the model, summarized in Table 1 [5, 6, 39, 40, 43–48]. Any information from a study in the Northeastern US that was not used to estimate a parameter directly was used instead to compare with model outputs to make sure the model was producing reasonable seasonal patterns of tick populations, on-host tick distributions, and host densities. Seasonal effects produced by the model could be compared to the range observed in multiple studies. The range of hosts considered allows us to compare model predictions with measured tick burdens and disease prevalence on a variety of hosts in several published field studies in the Northeast [21, 32–35, 49–53]. Across all of these measures, the model produces results that are biologically reasonable compared to what are observed. The model produces a synthetic ecosystem and epidemiology that is the basis for numerical experiments.

To study the role of increased mean annual temperature on vector dynamics and disease prevalence, a daily seasonal cycle for Hanover, NH, was fitted by a truncated Fourier series and the constant term in that series was increased. It is assumed that other variables such as host distribution remained constant.

**Figure 1:**
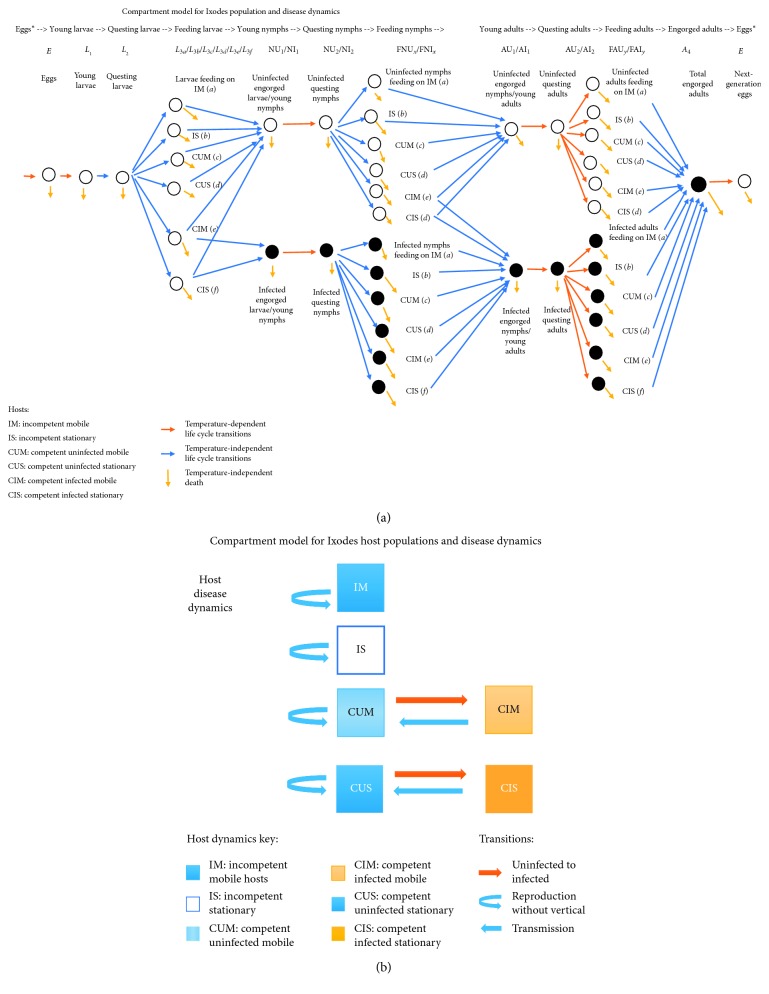
Compartment model for *I. scapularis* and host population and disease dynamics. (a) The life cycle of *I. scapularis* as described by equations (1)–(17). Feeding populations are split according to host type. Temperature-dependent maturation transitions are indicated in orange. (b) Host population and disease dynamics. Disease transitions require vector populations shown in this figure.

Figures 1(a) (ticks) and 1(b) (hosts) offer an overview of the relationships modeled in this study.  Figure 1(a) tracks the tick life cycle. During periods of feeding, the populations are split into six categories depending on the host. The potential of contracting disease from an infected host leads to twelve tick categories at the “feeding nymph” and “feeding adult” stages.  Figure 1(b) tracks transitions in the six host compartments. We assume there is no vertical transmission in either the tick or host life cycle.

Host species listed in Levi et al. [39] were categorized based on their reservoir competencies and whether or not a species was (relatively) mobile. All species listed in Levi et al. were categorized as follows:

1. Competent stationary hosts: white footed mouse, eastern chipmunk, masked shrew, short tailed shrew, eastern gray squirrel
2. Competent mobile hosts: ground foraging birds
3. Incompetent stationary hosts: striped skunk, raccoon, Virginia opossum
4. Incompetent mobile hosts: white-tailed deer

The ability of hosts to actively remove ticks is reflected in the per host carrying capacities. For simplicity, the model embraces a form of the null hypothesis, by assuming that tick-host contact rates depend on population densities of hosts and ticks irrespective of life stage and that all on-host ticks taken together are subject to a single on-host carrying capacity.

**Figure 2:**
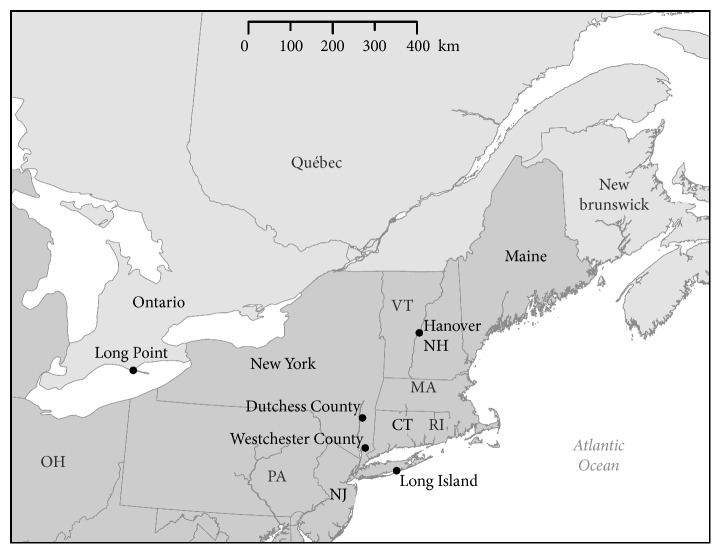
Map of Northeastern United States. Most field studies considered are from this region. Locations referenced in the text are labelled in black.

Simulations were run on a 2015 MacBook Pro using Matlab ode45 solver [36], with run times of approximately 8 seconds.  Figure 2 shows a map of the Northeastern United States, where most of the field studies cited were done.

### 2.1. Equations

The two-year life cycle for *I. scapularis* was broken into differential equations based on behaviorally distinguishable life stages as follows: eggs, young larvae, questing larvae, feeding larvae, engorged larvae, questing nymphs, feeding nymphs, engorged nymphs, questing adults, feeding adults, and engorged adults.

#### 2.1.1. Temperature (*T*)

The mean seasonal temperature cycle for Hanover, NH, was calculated from daily maximum and minimum temperature data obtained from the National Centers for Environmental Information (NCEI) Global Historical Climatology Network (GHCN) dataset.

GHCND data from both the Hanover and the Storrs, CT stations were used to create a seasonal average (1990–2015, Patch_Lyme) [54–56]. The data are modeled by a continuous function for temperature based on the day of the year for the model using a five-term Fourier series:

(1) $$\begin{array}{c} T=10.5+w+1\;*\;\left(\left(-10.79\right)\;\cos\left(t\;*\;0.0172\right)+\left(-7.53\right)\;\sin\left(t\;*\;0.0172\right)+\left(-1.212\right)\cos\left(2\;*\;t\;*\;0.0172\right)+\left(-0.07472\right)\sin\left(2\;*\;t\;*\;0.0172\right)\right). \end{array}$$

The default value for *w* is zero, corresponding to current temperature data from Hanover. In numerical experiments, *w* is increased in increments of 1°C, which effectively increases the temperature at every time step and therefore the annually averaged mean temperature. While the actual seasonal pattern of anthropogenic temperature change will be different from the simple additive shift used in this study, the magnitude of warming is consistent with the anticipated range of increasing temperatures for the Northeast projected by global climate models: 2.2°C for midcentury and intermediate greenhouse gas emissions to 6.4°C for late-century and high greenhouse gas emissions [57].

The maturation of eggs to larvae is based on the laboratory studies by Ogden et al. [6]. In this study, maturation times are given for a selection of warm temperatures. These maturation times were converted to rates and fit with a Gaussian distribution. To ensure diapause at low enough temperatures, a Heaviside function was incorporated that reduces maturation rates to zero when the temperature is below 15-degree Celsius. Combining these gives the temperature-dependent egg maturation function, *m*_e_(*T*), where HS is the Heaviside function:

(2a) $$\begin{array}{c} \;m_{\text{e}}\left(T\right)=0.0552\;*\;\exp\left(-{\left(\frac{T-25.83}{4.946}\right)}^{2}\right)\;*\text{ HS}\left(T-15\right). \end{array}$$

Based on [6] and using the same method as for *m*_e_(*T*), engorged larva matures with a temperature-dependent rate:

(2b) $$\begin{array}{c} \;m_{n1}\left(T\right)=m_{n1}\;*\;0.04001\;*\;\exp\left(-{\left(\frac{T-26.68}{9.533}\right)}^{2}\right)\;*\text{ HS}\left(T-15\right). \end{array}$$

Based on [6] and using the same method as for *m*_e_(*T*), engorged nymphs have a temperature-dependent maturation rate:

(2c) $$\begin{array}{c} m_{A1}\left(T\right)=0.03173\;*\;\exp\left(-{\left(\frac{T-25.83}{9.042}\right)}^{2}\right)\;*\text{ HS}\left(T-15\right). \end{array}$$

These temperature dependencies give rise to the changing tick and disease dynamics in this study.

#### 2.1.2. Tick Dynamics

Tick dynamics include maturation into and out of each stage as well as a death rate. On host, stages are divided according to the disease status of both hosts and ticks. Quantities tracked include eggs (*E*), young hardening larvae (*L*_1_), questing larvae (*L*_2_), larvae feeding on host type *x* (*L*_3*x*_), as there are six types of host (IM, IS, CUM, CUS, CIM, and CIS) which for convenience we relabel (*U*_*a*_, *U*_*b*_,…, *U*_*f*_) and set *x* as an index for *a*–*f*. Equations for uninfected engorged larvae (NU_1_) and infected engorged larvae (NI_1_) include disease transmission terms based on tick/host interactions. Uninfected and infected questing nymphs (NU_2_ and NI_2_) and feeding nymphs (FNU_*x*_ and FNI_*x*_) are tracked similar to larvae. Uninfected and infected engorged nymphs (AU_1_ and AI_1_) include disease transmission terms similar to those for engorged larvae. The equations for uninfected and infected questing and feeding adults (AU_2_, AI_2_, AU_3*x*_, and AI_3*x*_) are constructed similarly to those for nymphs and larvae. Engorged adults (*A*_4_) complete the life cycle:

(3) $$\begin{array}{c} E^{\prime }=\;b\;*\;A_{4}-\;d_{\text{e}}\;*\;E-\;m_{\text{e}}\left(T\right)\;*\;E, \end{array}$$

(4) $$\begin{array}{c} L_{1}^{\prime }=\;m_{\text{e}}\left(T\right)\;*\;E-\;d_{1}\;*\;L_{1}-\;m_{1}\;*\;L_{1}, \end{array}$$

(5) $$\begin{array}{c} L_{2}^{\prime }=\;m_{1}\;*\;L_{1}-\;d_{2}\;*\;L_{2}-\;m_{2}\;*\;L_{2}, \end{array}$$

(6) $$\begin{array}{c} L_{3x}^{\prime }=m_{2}\;*\;Q_{x}\;*\;L_{2}\;*\;F_{x}-\;d_{3x}\;*\;L_{3x}-m_{3}\;*\;L_{3x}. \end{array}$$

The first term represents attachment to a host where the parameter *m*_*2*_ is a constant rate based on the estimated number of days spent questing, *Q*_*x*_ is the probability that the host is of type *x* (described in equation 21), and *L*_2_ the population of questing larvae. The index *F*_*x*_ (described in equation 22) is a function of total number of hosts of type *x*, the per host carrying capacity for ticks, and the amount of that capacity already occupied by ticks. *F*_*x*_ is nonlinear and caps the feeding larvae population in terms of hosts:

(7) $$\begin{array}{c} {\text{NU}}_{1}^{\prime }=\;m_{3a}L_{3a}+\;m_{3b}L_{3b}+\;m_{3c}L_{3c}+\;m_{3d}L_{3d}+\left(1-p_{L}\right)\left(m_{3e}L_{3e}+m_{3f}L_{3f}\right)-\;d_{n1}{\text{NU}}_{1}-\;m_{n1}\left(T\right){\text{NU}}_{1}, \end{array}$$

(8) $$\begin{array}{c} {\text{NI}}_{1}^{\prime }=\;p_{L}\left(m_{3e}L_{3e}+m_{3f}L_{3f}\right)\;-\;d_{n1}{\text{NI}}_{1}-\;m_{n1}\left(T\right){\text{NI}}_{1}, \end{array}$$

(9) $$\begin{array}{c} {\text{NU}}_{2}^{\prime }=\;m_{n1}\left(T\right){\text{NU}}_{1}-d_{n2}{\text{NU}}_{2}-\;m_{n2}{\text{NU}}_{2}, \end{array}$$

(10) $$\begin{array}{c} {\text{NI}}_{2}^{\prime }=m_{n1}\left(T\right){\text{NI}}_{1}-d_{n2}{\text{NI}}_{2}-m_{n2}{\text{NI}}_{2}, \end{array}$$

(11) $$\begin{array}{c} {\text{FNU}}_{x}^{\prime }=m_{n2}{\text{NU}}_{2}F_{x}Q_{x}-d_{fnx}{\text{FNU}}_{x}-m_{fn}{\text{FNU}}_{x}, \end{array}$$

(12) $$\begin{array}{c} {\text{FNI}}_{x}^{\prime }=m_{n2}{\text{NI}}_{2}F_{x}Q_{x}-d_{fnx}{\text{FNI}}_{x}-m_{fn}{\text{FNI}}_{x}, \end{array}$$

(13) $$\begin{array}{c} {\text{AU}}_{1}^{\prime }=m_{fn}\left({\text{FNU}}_{a}+{\text{FNU}}_{b}+{\text{FNU}}_{c}+{\text{FNU}}_{d}\right)+m_{n3}\left(1-p_{n}\right)\left({\text{FNU}}_{e}+{\text{FNU}}_{f}\right)-d_{A1}{\text{AU}}_{1}-m_{A1}\left(T\right){\text{AU}}_{1}, \end{array}$$

(14) $$\begin{array}{c} {\text{AI}}_{1}^{\prime }=m_{fn}\left({\text{FNI}}_{a}+{\text{FNI}}_{b}+{\text{FNI}}_{c}+{\text{FNI}}_{d}\right)+\;m_{n3}\left(p_{n}\right)\left({\text{FNU}}_{e}+{\text{FNU}}_{f}\right)-d_{A1}{\text{AI}}_{1}-m_{A1}\left(T\right){\text{AI}}_{1}, \end{array}$$

(15) $$\begin{array}{c} {\text{AU}}_{2}^{\prime }=m_{A1}\left(T\right){\text{AU}}_{1}-d_{A2}{\text{AU}}_{2}-m_{A2}{\text{AI}}_{2}, \end{array}$$

(16) $$\begin{array}{c} {\text{AI}}_{2}^{\prime }=m_{A1}\left(T\right){\text{AI}}_{1}-d_{A2}{\text{AI}}_{2}-m_{A2}{\text{AI}}_{2}, \end{array}$$

(17) $$\begin{array}{c} {\text{AU}}_{3x}^{\prime }=m_{A2}{\text{AU}}_{2}F_{x}Q_{x}-d_{A3x}{\text{AU}}_{3x}-m_{A3}{\text{AU}}_{3x}, \end{array}$$

(18) $$\begin{array}{c} {\text{AI}}_{3x}^{\prime }=m_{A2}{\text{AI}}_{2}F_{x}Q_{x}-d_{A3}{\text{AI}}_{3x}-m_{A3}{\text{AI}}_{3x}, \end{array}$$

(19) $$\begin{array}{c} A_{4}^{\prime }=m_{A3}{\text{AU}}_{3\text{total}}+m_{A3}{\text{AI}}_{3\text{total}}-d_{A4}A_{4}. \end{array}$$

#### 2.1.3. Host Dynamics

Host population dynamics are represented by logistic growth with observed per km^2^ densities and death rates. Disease dynamics are given in terms of encounter rates with infected feeding ticks. Quantities tracked are incompetent mobile hosts (IM), incompetent stationary hosts (IS), competent mobile hosts both uninfected and infected (CUM and CIM), and competent stationary hosts both uninfected and infected (CUS and CIS):

(20) $$\begin{array}{c} {\text{IM}}^{\prime }=\;b_{\text{IM}}\text{IM}\left(1-\frac{\text{IM}}{K_{\text{IM}}}\right)-\;d_{\text{IM}}\text{IM}, \end{array}$$

(21) $$\begin{array}{c} {\text{IS}}^{\prime }=b_{\text{IS }}\text{IS}\left(1-\frac{\text{IS}}{K_{\text{IS}}}\right)-\;d_{\text{IS}}\text{IS,} \end{array}$$

(22) $$\begin{array}{c} {\text{CUM}}^{\prime }=b_{\text{CUM}}\left(\text{CUM}+\text{CIM}\right)\left(1-\frac{\text{CUM}+\text{CIM}}{K_{\text{CM}}}\right)-\;d_{\text{CUM}}\text{CUM}-\;p_{\text{CUM}}\left({\text{FNI}}_{c}+\text{AI}3_{c}\right)\;*\text{ CUM,} \end{array}$$

(23) $$\begin{array}{c} {\text{CUS}}^{\prime }=b_{\text{CUS}}\left(\text{CUS}+\text{CIS}\right)\left(1-\frac{\text{CUS}+\text{CIS}}{K_{\text{CS}}}\right)-\;d_{\text{CUS}}\text{CUS}-p_{\text{CUS}}\left({\text{FNI}}_{d}+{\text{AI}3}_{d}\right)\;*\text{ CUS,} \end{array}$$

(24) $$\begin{array}{c} {\text{CIM}}^{\prime }=p_{\text{CUM}}\left({\text{FNI}}_{c}+\text{AI}3_{c}\right)\;*\text{ CUM}-d_{\text{CIM}}\text{CIM,} \end{array}$$

(25) $$\begin{array}{c} {\text{CIS}}^{\prime }=p_{\text{CUS}}\left({\text{FNI}}_{d}+\text{AI}{3\;}_{d}\right)\;*\text{ CUS}-d_{\text{CIS}}\text{CIS.} \end{array}$$

#### 2.1.4. Auxiliary Equations

Recall that there are six types of host (IM, IS, CUM, CUS, CIM, CIS) which for convenience we relabel (*U*_*a*_, *U*_*b*_,…, *U*_*f*_) and set *x* as an index for *a*–*f*.

To count total ticks on a given type of host, we have:

- Larvae *L*_3*x*_ where *x* = *a*, *b*, *c*, *d*, *e*, *f*, feeding on hosts of type *x*
- Uninfected nymphs feeding on hosts of type *x*, denoted FNU_*x*_
- Infected nymphs feeding on hosts of type *x*, denoted FNI_*x*_
- Uninfected adults feeding on hosts of type *x*, denoted AU_3*x*_
- Infected adults feeding on hosts of type *x*, denoted AI_3*x*_ where *x* = *a*, *b*, *c*, *d*, *e*, *f*

Let *T*_*x*_ be the total ticks on hosts of type *x*, so *T*_*x*_ = *L*_3*x*_ + FNU_*x*_ + FNI_*x*_ + AU_3*x*_ + AI_3*x*_.

Let *C*_*x*_ be the per host carrying capacity for ticks on a host of type *x*.

Let *S* = IM + IS + CUM + CUS + CIM + CIS be the total number of hosts of all types.

For each respective host type we have the following equations:

(26) $$\begin{array}{c} Q_{x}=\frac{U_{x}}{\left(S+r\right)}. \end{array}$$

Equation (26) expresses the approximate fraction of hosts that are of type *x*. The parameter *r* is set to a small number to avoid numerical issues if the number of hosts is set close to zero:

(27) $$\begin{array}{c} F_{x}=\max\left(\frac{C_{x}\;*\;U_{x}-T_{x}}{C_{x}\;*\;U_{x}+r},\;0\right). \end{array}$$

Equation (27) describes the available on-host space for ticks on hosts that are of type *x*. *T*_*x*_ counts the number of ticks on hosts of type *x*. The term *C*_*x*_ *∗* *U*_*x*_ is the per host tick capacity times the total number of hosts of type *x*, giving the per host tick capacity for hosts of type *x*. The difference between these two is the available space for ticks on all hosts of type *x*.

### 2.2. Numerical Simulations

Simulations were run on Matlab software [36] using the ODE45 solver for a ten-year simulated time period. The model achieved a periodic steady state before the last year, which is the year used for all figures and calculations. Parameters and initial conditions are listed in Table 1.

## 3. Results

**Figure 3:**
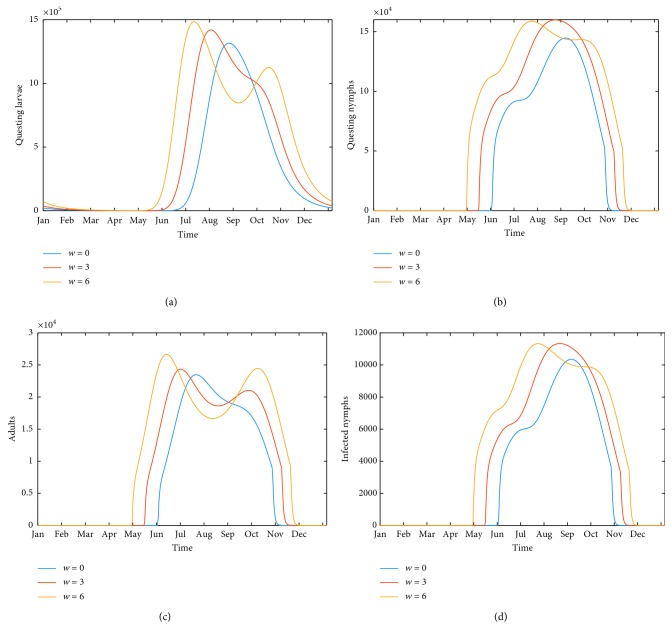
Temporal dynamics of tick populations and selected stages at steady state for historical temperature, 3°C above historical, and 6°C above historical. (a) Seasonal pattern of questing larvae (*L*_2_), (b) questing nymphs (NU_2_ + NI_2_), (c) adults (AU_2_ + AI_2_), and (d) infected nymphs (NI_2_) per km^2^. The time axis represents the final year of the run from Jan 1 to Dec 31. Note the difference in scale of the four panels.

**Figure 4:**
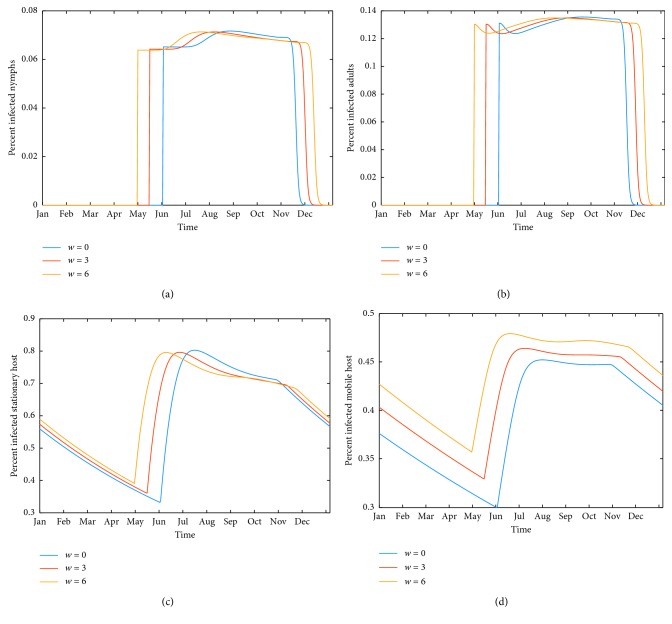
Temporal dynamics of Lyme disease. Temporal dynamics of *Borrelia burgdorferi* infection at steady state for historical temperature, 3°C above historical, and 6°C above historical. (a) *B. burgdorferi* prevalence in questing nymphs (NI_2_/(NI_2_ + NU_2_) (b) *B. burgdorferi* presence in questing adult ticks (AI_2_/(AI_2_ + AU_2_). (c) *B. burgdorferi* prevalence in competent stationary hosts (CIS/(CIS + CUS)) (d) *B. burgdorferi* presence in competent mobile hosts (CIM/(CIM + CUM)). Note the difference in scale of the four panels.

**Figure 5:**
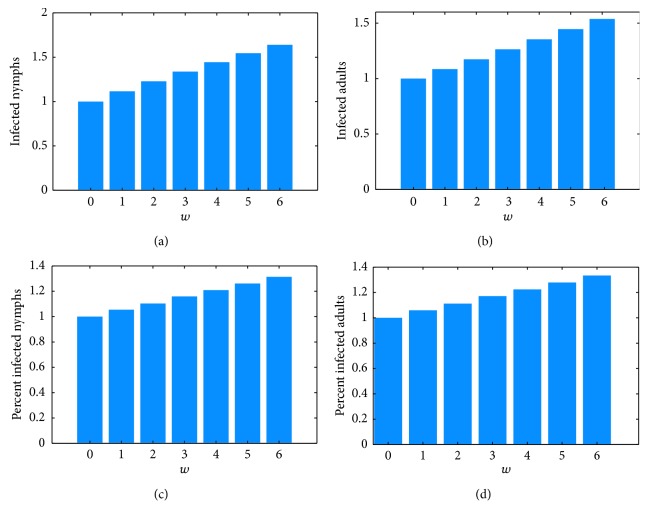
Response of tick-based risk measures to increasing temperature, as percent of control (*w* = 0, historical temperature). (a) Number of infected nymphs per day averaged over the last year of the simulation, scaled by the same quantity for *w* = 0 (no temperature rise), i.e., av(NI_2_)_*w*=*i*_/av(NI_2_)_*w*=0_, *i*=0,1,…, 6. (b) Number of infected adults per day (averaged and scaled to *w* = 0), i.e., av(NA_2_)_*w*=*i*_/av(NA_2_)_*w*=0_, *i*=0,1,…, 6. (c) Percent nymphs infected (averaged and scaled to *w* = 0), i.e., av(NI_2_/(NU_2_+NI_2_))_*w*=*i*_/av(NI_2_/(NU_2_+NI_2_))_*w*=0_, *i*=0,  1,…, 6. (d) Percent adults infected (averaged and scaled to *w* = 0), i.e., av(AI_2_/(AU_2_+AI_2_))_*w*=*i*_/av(AI_2_/(AU_2_+AI_2_))_*w*=0_, *i*=0,  1,…, 6.

**Figure 6:**
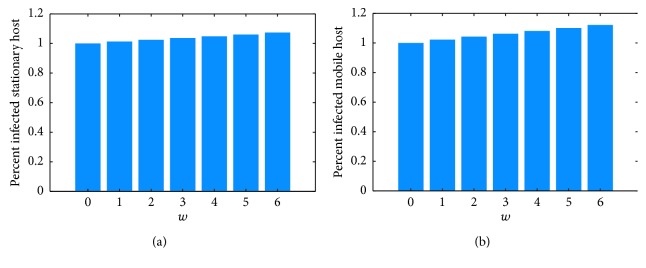
Response of host-based risk measures to increasing temperature, as percent of control (no temperature rise). (a) Percent infected competent stationary hosts per day averaged over the last year of the simulation, scaled by the same quantity for *w* = 0 (no temperature rise), i.e., av (CIS/(CUS+CIS))_*w*=*i*_/av(CIS/(CUS+CIS))_*w*=0_, *i*=0,  1,…, 6 (b) Percent infected competent mobile hosts per day averaged over the last year of the simulation, scaled by the same quantity for *w* = 0 (no temperature rise), i.e., av (CIM/(CUM+CIM))_*w*=*i*_/av(CIM/(CUM+CIM))_*w*=0_, *i*=0,  1,…, 6.

**Figure 7:**
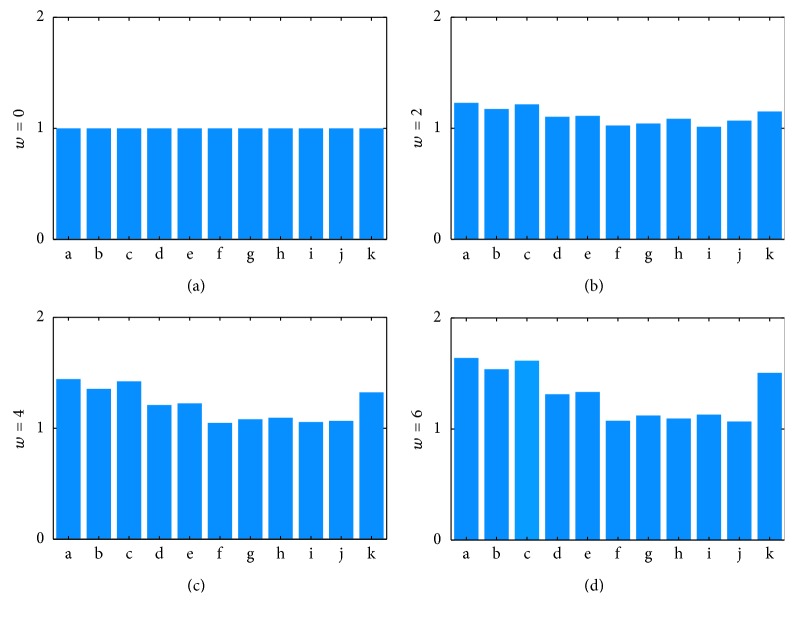
Response of various risk measures to increasing temperatures (relative to historical temperature, *w* = 0) for 0, 2, 4, and 6°C temperature increase. Each risk measure is scaled against its own value at *w* = 0. In this figure, a, b, c, d, e, f, g, h, i, j, and k represent the following: (a) annual average of number of questing nymphs (NI_2_) at steady state, (b) annual average of infected questing adult ticks (AI_2_) at steady state, (c) annual average of infected questing ticks (AI_2_ + NI_2_) at steady state, (d) annual average of disease prevalence in questing nymphs (NI_2_/(NI_2_ + NU_2_)) at steady state, (e) annual average of disease prevalence in questing adult ticks (AI_2_/(AI_2_ + AU_2_)) at steady state, (f) annual average of disease prevalence in competent stationary hosts (CIS/(CIS + CUS)) at steady state, (g) annual average of disease prevalence in competent mobile hosts (CIM/(CIM + CUM)) at steady state, (h) maximum daily number of infected nymphs (NI_2_) at steady state, (i) maximum daily number of infected adult ticks (AI_2_) at steady state, (j) maximum daily number of infected ticks (AI_2_ + NI_2_) at steady state, and (k) degree days at steady state.

Overall model performance is compared with field observations from the Northeastern US, showing the model produces reasonable results. The results of numerical experiments increasing mean annual temperature are shown Figures 3–7 and discussed.

### 3.1. Overall Model Performance

Counts of questing larva in Dutchess County, NY, 1992–1994, were observed to sometimes have unimodal distributions peaking anywhere from early July to early August and bimodal distributions with peaks in June and again in August. Questing nymphs were observed to have a unimodal peak during June/July. Adults were more likely to have a bimodal distribution, peaking as early as May and as late as November. Years with unimodal questing adult distributions peaked late, in October [50, 51]. Estimates of average questing larva density varied from 1.45 to 3.20 per square meter depending upon study site. Estimates of average nymph density ranged from 0.07 to 0.32 per square meter. The model predicts peak questing larva counts of 1.46 per m^2^ near Aug 19 and peak questing nymph counts of 0.146 per m^2^ near Aug 30, reasonably close to the field observations described above, although no attempt was made to match those data.

Studies also report an average tick burden for mice, *P. leucopus*, as 10–15 in a Dutchess County, NY, study for 1991–1993 [50]. A subsequent study in the same region in 1995, 1997, and 1998 reports a larva burden of 6 on mice and 17 on chipmunks, and a nymph burden of 5 on mice and 2 on chipmunks [52]. The annual mean maximum larva per rodent was reported in the Long Point Ontario data for 1990 and 1992 as 10–32, with feeding nymph to feeding larva ratio of 0.15 [53]. The Levi et al. study from which we derived many of the model parameters presents a synopsis of 19 years of data from Dutchess County, NY, showing that peak larva to mouse ratios can range from less than 10 to over 40, while peak nymph to mouse ratios range from 1 to 4 [39]. In our model, chipmunks, mice, shrews, and squirrels are all grouped together as “competent stationary hosts,” for which it predicts a maximum tick to host ratio of around 26 as seen in Figure 3(d), in reasonable agreement with these studies.

Long Point, Ontario, data for 1989–1992 also report average total tick per deer between 170 and 249 [53]. The model here gives a maximum of about 55 ticks per deer, which is lower than numbers reported in this study. However, the peak tick per competent mobile host is about 10, very close to the per host carrying capacity for that category. The ratio for incompetent stationary hosts, 40, was also close to the per host carrying capacity for that category of host. Discrepancies could be explained by differing host distributions.

The field data present a range of observations that reflect uncertainty in the expected timing of emergence and on-host tick burdens. In addition, no data set has complete information on host populations. The conclusion from these comparisons is that, although there appear to be some discrepancies in the timing of peak questing nymphs and peak tick burden on deer, the overall results of the model are within the ranges observed across the field studies considered and the model is good enough to test the progression of disease and the impact of changes in host populations on both tick populations and disease.

The literature includes studies of disease prevalence in both ticks and hosts. Prevalence changes greatly from location to location and over time. In earlier studies measuring disease prevalence in ticks, the range goes from 3% infected [21] to 24% [32] and to 33% [33]. The Stafford study [32] indicates a seasonal trend with prevalence varying from 8% to 24%. Studies of *B. burgdorferi* in mice also report a seasonal trend, from 33% to 75% [34] to 57% to 93% [35]. Disease prevalence in the model has a seasonal trend similar to these.

### 3.2. Results of Numerical Simulations

The model presented here produces a periodic seasonal cycle of tick prevalence, as observed in many locations, while host populations all arrive and remain at equilibrium. As the temperature input is strictly periodic, there is no variation from year to year after an initial transient period. The behavior of questing tick populations over a typical steady state year is shown in Figures 3(a) (historical temperature), 3(b) (3°C above historical), and 3(c) (6°C above historical). Similarly, the model produces seasonal patterns of infected questing nymphs, shown in Figure 3(d), for the same three temperature patterns. Both the length of the questing season and the number of infected questing ticks are seen to increase with temperature. Figures 4(a) and 4(b) show changes in disease prevalence in questing nymphs and adults, showing a longer season of potential transmission to humans with rising temperature. Figures 4(c) and 4(d) show changes in disease prevalence in host populations. Although a seasonal trend is still visible, the absolute change in numbers of infected hosts is small due to longer lifespans.

The response of tick-based risk measures to increasing temperature, as percent of control under temperature increases from 1°C to 6°C by increments of 1°C, is shown in Figure 5. Comparing Figures 5(a) and 5(b) show that the number of infected questing nymphs rises faster with increased temperature than the number of infected questing adults. Figures 5(c) and 5(d) show the disease prevalence in nymphs also rises faster than in adults. The model predicts over five times as many questing nymphs as adults, as seen in Figure 3. Together, these explain why the number of infected questing nymphs is close to the total number of infected questing ticks with rising temperature, as shown in Figure 7.

Risk measures based on disease prevalence in hosts are shown in Figure 6. Average annual prevalence in stationary hosts barely rises at all (Figure 6(a)). From Figure 4(c), one can see that the small rise that is visible is due to the longer season of transmission rather than to a larger percentage of infected hosts.  Figure 6(b) shows a slightly higher effect on competent mobile hosts, due to both a longer season and slightly higher prevalence, shown in Figure 4(d). These hosts are birds, which can transmit the disease across long distances.

Risk based on degree days is not shown in Figure 6 but is included in Figure 7, which compares the measures of risk against each other for a given rise in temperature. Figure 7 compares each possible measure of risk against its value at *w* = 0. Bar c in each chart represents how the average number of infected questing ticks changes with rising temperature. Each bar may be compared to bar c to gauge how well that measure tracks the average number of infected questing ticks.

## 4. Discussion

While it is unreasonable to expect a model of a hypothetical but biologically reasonable scenario to match any particular study, we highlight places where the model could potentially be improved. The increase in mean annual temperature as a driver of increased risk of Lyme disease is discussed. The many possible measures of risk are compared to the intuitively solid criterion of average infected questing tick prevalence.

### 4.1. Potential Model Improvements

One explanation for the discrepancy between observed and predicted tick burden on deer is that the model allocates questing ticks according to the fraction of hosts in a category, and the number of incompetent mobile hosts (deer) is very low compared to the other categories. A questing tick is thus far more likely to find a different host. Those other hosts are effectively decoys. Another possibility is that deer are more prevalent in Dutchess County, NY, than estimates from [39] suggest, increasing the probability of encounter. The model currently assumes that ticks choose a host based on the probability of encounter measured by relative density of a particular host type, but perhaps, this is not entirely true. It is possible that larvae are in locations where small mammals are more likely to be present, and are more likely to be on these, while adult ticks are higher in the brush and more likely to encounter deer. A survey of feeding ticks on hosts of various types according to their life stage could shed light on this question and possibly offer an electivity index [58, 59] for each stage of questing tick, as has been done for other organisms [60].

### 4.2. Effect of Rising Mean Annual Temperature

We find that increasing temperatures result in a higher risk of Lyme disease. Figure 3 shows that an overall increase in the number of ticks is due more to the longer season of activity, as peak counts do not differ much from one temperature to the next. Similarly, Figures 4(a) and 4(b) show similar peak prevalence of disease in questing ticks regardless of temperature increase, with an overall increase of average prevalence with warmer temperatures due to the extended season.

Figure 5 supports this, showing an average increase in disease prevalence in questing adults and nymphs of 30% for a 6°C temperature increase (Figures 5(c) and 5(d)). The rise in overall numbers of infected ticks is greater, an almost 50% increase for a 6°C temperature increase (Figures 5(a) and 5(b)). However, a field measurement taken at midsummer would show approximately the same population density of infected questing ticks for all mean annual temperatures. The model suggests that increased risk from warmer temperatures comes from somewhat higher tick populations (seen in Figure 4) coupled with a longer season of transmission (seen in Figures 5(a) and 5(b)).

Figure 6 shows a small increase in prevalence among host populations.  Figure 4(b) indicates that for the competent stationary hosts, the transmission cycle starts earlier in the season but the peak does not increase much. The dynamics are somewhat different for the competent mobile hosts. For these, the season is longer and the peak is a bit higher (Figure 4(c)). Host prevalence measures show only around 10% increase over the 6-degree rise in temperature (Figures 6(a) and 6(b)).

### 4.3. Comparing Risk Measures

Figure 7 summarizes the relative performance of these various measures of risk compared to the intuitively clear measure of abundance of infected questing ticks (NI_2_ + AI_2_) integrated over the course of the year. Figure 7 shows that overall abundance of infected ticks (bar a in the figure) rises with temperature more quickly than the usual field measurements would indicate. Increased risk of disease for humans is likely to depend on this abundance, which increases with temperature more than disease prevalence in hosts or ticks alone would indicate. Figure 7 in particular indicates that the response of degree days to rising temperature tracks the rising abundance of infected questing ticks better than prevalence measures.

This study does not include human behavior that affects the likelihood of encounter with infected ticks. Overall abundance is a reasonable measure for humans spending time outdoors over a long season, for example, through-hikers on the Appalachian Trail or rural populations. Overall abundance of infected ticks increases by about 50% for this period (Figures 5(a) and 5(b)), largely due to the extended tick season. It is less important for children on a short summer vacation in the country during high tick season, where maximum abundance of infected ticks describes local contact during that period, and which does not vary much with rising temperature (Figure 7).

## 5. Conclusions

The model of tick populations and Lyme disease developed in this study, based on earlier work by Ogden et al. [5, 6], incorporates temperature-dependent maturation rates and six categories of tick host populations based on whether they are incompetent or competent Lyme disease hosts, whether they are infected or uninfected, and whether they are relatively mobile or stationary. The results of the model were compared with field data taken from a variety of studies in the Northeast US. The model produces, on the whole, biologically reasonable results, including the seasonality of tick populations, observed on-host tick burdens, and disease prevalence at steady state.

The model predicts rising risk of Lyme disease with increasing temperature although not all risk measures give the same enhanced increased risk for a given temperature change. Compared to the intuitively clear measure of infected tick abundance, more easily measured quantities such as overall disease prevalence in ticks or hosts rise relatively slowly. Measures of disease prevalence in ticks or hosts at a single time point barely change at all with rising temperature during the high transmission season. These measures are therefore likely to give a lower estimate of increased risk to humans than is the case. Other than absolute measures of infected tick abundance, the best measure of overall risk was degree days greater than zero. For shorter visits to tick-infested areas during the high transmission season, risk would be measured by maximum abundance of infected ticks, which does not change much with temperature.

## Figures and Tables

**Table 1:** Default parameters used in model.

Parameter	Meaning	Value	Source
*w*	Increase in mean annual temperature relative to historical	0- to 5-degree Celsius	Arbitrary
*b*	Per capita egg production per day	300	[5, 6]
*d*_e_	Daily death rate for eggs	0.015	[43]
*d*_1_	Daily death rate of young hardening larvae	0.01	[44]
*d*_*n*1_, *d*_*A*1_	Daily death rate of engorged larvae and engorged nymphs	0.001	[45]
*d*_2_, *d*_*n*2_, *d*_*A*2_	Daily death rate of questing larvae, nymphs, and adults	0.094	[44]
*d*_3*a*_ = *d*_*fna*_ = *d*_*A*3*a*_	Daily death rate of feeding larva, nymphs, and adults on host type *a* (IM)	0.51	[39]
*d*_3*b*_ = *d*_*fnb*_ = *d*_*A*3*b*_	Daily death rate of feeding larva, nymphs, and adults on host type *b* (IS)	0.89	[39]
*d*_3*c*_ = *d*_*fnc*_ = *d*_*A*3*c*_ = *d*_3*e*_ = *d*_*fne*_ = *d*_*A*3*e*_	Daily death rate of feeding larva, nymphs, and adults on host types *c* and *e* (CUM, CIM)	0.73	[39]
*d*_3*d*_ = *d*_*fnd*_ = *d*_*A*3*d*_ = *d*_3*f*_ = *d*_*fnf*_ = *d*_*A*3*f*_	Daily death rate of feeding larva, nymphs, and adults on host types *c* and *e* (CUS, CIS)	0.72	[39]
*d*_*A*4_	Daily death rate of engorged adults	0.5	[5, 6]
*m*_e_(*T*)	Egg to larvae maturation rate	*m*_e_(*T*)= 0.0552 *∗* exp(−((*T* − 25.83)/4.946)^2^) *∗* HS(*T* − 15)	[6]
*m*_1_	Young hardening larvae to questing larvae maturation rate	0.033	[43]
*m*_2_, *m*_*n*2_, *m*_*A*2_	Questing tick to feeding tick maturation (all stages)	0.5	Estimated questing period of 5–6 days
*m*_3_, *m*_*fn*_, *m*_*A*3_	Feeding tick maturation rate, all stages, all hosts	0.5	[46]
*m*_*n*1_(*T*)	Engorged larvae maturation rate	*m*_*n*1_(*T*)= 0.04001*∗* exp(−((*T* − 26.68)/9.533)^2^) *∗* HS(*T* − 15)	[6]
*m*_*A*1_(*T*)	Engorged nymph maturation rate	*m*_*A*1_(*T*)= 0.03173 *∗* exp(−((*T* − 23.85)/9.042)^2^) *∗* HS(*T* − 15)	[6]
*r*	A numerical feature to ensure you never divide by 0	0.001	
*p*_*L*_	Probability of larvae infection	0.1	Estimated
*p*_*N*_	Probability of nymph infection	0.1	Estimated
*C*_*a*_	Per host tick carrying capacity of host type *a*, incompetent mobile	239	[39]
*C*_*b*_	Per host tick carrying capacity of incompetent stationary hosts	176.75	[39]
*C*_*c*_ = *C*_*e*_	Per host tick carrying capacity of CUM and CIM hosts	11.4	[39]
*C*_*d*_ = *C*_*f*_	Per host tick carrying capacity of CUS and CIS hosts	46.84	[39]
*b*_IM_	Birth rate of incompetent mobile hosts	0.00261	[39, 40, 47]
*b*_IS_	Birth rate of incompetent stationary hosts	0.0102	[39, 40, 47]
*b*_CUM_	Birth rate of competent uninfected mobile hosts	0.00753	[39, 40, 48]
*b*_CUS_	Birth rate of competent uninfected stationary host	0.0176	[39, 40, 47]
*d*_IM_	Death rate of IM hosts	0.000609	[39, 40, 47]
*d*_IS_	Death rate of IS hosts	0.00129	[39, 40, 47]
*d*_CUM_ = *d*_CIM_	Death rate of competent uninfected mobile hosts	0.00157	[39, 40, 48]
*d*_CUS_ = *d*_CIS_	Death rate of competent uninfected stationary hosts	0.00345	[39, 40, 47]
*K*_IM_	Cell carrying capacity of IM host	25	[39]
*K*_IS_	Cell carrying capacity of IS hosts	45	[39]
*K*_CM_	Cell carrying capacity of CUM + CIS hosts	3100	[39]
*K*_CS_	Cell carrying capacity of CUS + CIS hosts	9,335	[39]
*p*_CUM_	Probability of CUM host infection (per infective tick per day × number of hosts of that type)	0.117	[39]
*p*_CUS_	Probability of competent uninfected stationary host infection (per infective tick per day × number of hosts of that type)	0.6635	[39]
*E*(0)	Initial number of eggs	10,000,000	
NU_1_(0)	Initial uninfected engorged larvae	5,000,000	
AU_1_(0)	Initial uninfected engorged nymphs	300,000	
IM(0)	Initial incompetent mobile hosts	25	
IS(0)	Initial incompetent stationary hosts	45	
CUM(0)	Initial competent mobile hosts	3,100	
CUS(0)	Initial competent stationary hosts	9,335	
All other initial conditions		0	

## Data Availability

All data used in this study came from published, cited sources. The data used to support the findings of this study are available from the corresponding author upon request.
